# Biomarker potential of *ST6GALNAC3* and *ZNF660* promoter hypermethylation in prostate cancer tissue and liquid biopsies

**DOI:** 10.1002/1878-0261.12183

**Published:** 2018-03-13

**Authors:** Christa Haldrup, Anne L. Pedersen, Nadia Øgaard, Siri H. Strand, Søren Høyer, Michael Borre, Torben F. Ørntoft, Karina D. Sørensen

**Affiliations:** ^1^ Department of Molecular Medicine Aarhus University Hospital Denmark; ^2^ Department of Histopathology Aarhus University Hospital Denmark; ^3^ Department of Urology Aarhus University Hospital Denmark

**Keywords:** biomarker, epigenetics, liquid biopsy, prostate cancer, *ST6GALNAC3*, *ZNF660*

## Abstract

Current diagnostic and prognostic tools for prostate cancer (PC) are suboptimal, leading to overdiagnosis and overtreatment. Aberrant promoter hypermethylation of specific genes has been suggested as novel candidate biomarkers for PC that may improve diagnosis and prognosis. We here analyzed *ST6GALNAC3* and *ZNF660* promoter methylation in prostate tissues, and *ST6GALNAC3*,*ZNF660*,*CCDC181*, and *HAPLN3* promoter methylation in liquid biopsies. First, using four independent patient sample sets, including a total of 110 nonmalignant (NM) and 705 PC tissue samples, analyzed by methylation‐specific qPCR or methylation array, we found that hypermethylation of *ST6GALNAC3* and *ZNF660* was highly cancer‐specific with areas under the curve (AUC) of receiver operating characteristic (ROC) curve analysis of 0.917–0.995 and 0.846–0.903, respectively. Furthermore, *ZNF660* hypermethylation was significantly associated with biochemical recurrence in two radical prostatectomy (RP) cohorts of 158 and 392 patients and remained significant also in the subsets of patients with Gleason score ≤7 (univariate Cox regression and log‐rank tests, *P *<* *0.05), suggesting that *ZNF660* methylation analysis can potentially help to stratify low‐/intermediate‐grade PCs into indolent vs. more aggressive subtypes. Notably, *ZNF660* hypermethylation was also significantly associated with poor overall and PC‐specific survival in the RP cohort (*n *=* *158) with long clinical follow‐up available. Moreover, as proof of principle, we successfully detected highly PC‐specific hypermethylated circulating tumor DNA (ctDNA) for *ST6GALNAC3*,*ZNF660*,*HAPLN3*, and *CCDC181* in liquid biopsies (serum) from 27 patients with PC vs. 10 patients with BPH, using droplet digital methylation‐specific PCR analysis. Finally, we generated a three‐gene (*ST6GALNAC3*/*CCDC181*/*HAPLN3*) ctDNA hypermethylation model, which detected PC with 100% specificity and 67% sensitivity. In conclusion, we here for the first time demonstrate diagnostic biomarker potential of *ST6GALNAC3* and *ZNF660* methylation, as well as prognostic biomarker potential of *ZNF660*. Furthermore, we show that hypermethylation of four genes can be detected in ctDNA in liquid biopsies (serum) from patients with PC.

**Abbreviations**
AN: adjacent normal
AUC: area under the curve
BCR: biochemical recurrence
BPH: benign prostatic hyperplasia
cfDNA: cell‐free DNA
CRPC: castration‐resistant prostate cancer
CSS: cancer‐specific survival
ctDNA: circulating tumor DNA
ddMSP: methylation‐specific droplet digital PCR
ddPCR: droplet digital PCR
FF: fresh‐frozen
FFPE: formalin‐fixed paraffin‐embedded
HR: hazard ratio
MPC: metastatic prostate cancer
OS: overall survival
PC: prostate cancer
PIN: prostate intraepithelial neoplasia
PSA: serum prostate‐specific antigen
qMSP: quantitative methylation‐specific PCR
ROC analysis: receiver operating characteristic analysis
RP: radical prostatectomy
TCGA: The Cancer Genome Atlas
TURP: transurethral resection of the prostate

## 1 Introduction

Prostate cancer (PC) is the most common malignancy and the second leading cause of cancer‐related mortality among men in Western countries (Crawford, 2003). PC diagnosis is based on histopathological evaluation of prostate needle biopsies. Notably, the implementation in the 1990s of the serum prostate‐specific antigen (PSA) test as an initial indication for PC has led to a significant increase in PC incidence as well as in the number of biopsies performed. Prostate biopsy is associated with considerable risk of life‐threatening sepsis (Anderson *et al*., 2015; Toren *et al*., 2010), and a significant proportion of initial and repeat biopsies are negative for PC (Serag *et al*., 2012). Thus, better minimally invasive or noninvasive PC biomarkers that can replace or supplement PSA are needed to secure early and more accurate diagnosis and to reduce unnecessary prostate biopsies.

The majority of newly diagnosed PCs are localized and often of relatively low grade. Early diagnosis is pivotal for curative treatment, as organ‐confined PC can be cured by radical prostatectomy (RP) or radiation therapy, whereas only palliative treatments are available for metastatic PC (MPC). However, many patients with relatively indolent early‐stage PC will not benefit from surgery or radiation therapy (Hamdy *et al*., 2016). Indeed, a large number of patients with early‐stage PC are likely overdiagnosed and overtreated due to the limited accuracy of the currently used clinicopathological prognostic parameters (Salman *et al*., 2015). New emerging molecular biomarkers for PC aggressiveness have shown promising potential for improving risk stratification and thus might contribute to better and more individualized PC treatment in the future, but none are currently implemented in standard clinical practice (Pentyala *et al*., 2016).

Aberrant DNA hypermethylation of promoter‐associated CpG islands is characteristic for tumor cells, including PC cells (Jones and Laird, 1999; Baylin and Herman, 2000). Promoter hypermethylation is closely associated with gene silencing and may cause downregulation of, for example, tumor suppressor genes during carcinogenesis (Kulis and Esteller, 2010; Wu *et al*., 2015). Moreover, aberrant promoter hypermethylation of specific genes has been reported to hold promising diagnostic and/or prognostic biomarker potential for PC in tissue samples (Park, 2015; Sorensen *et al*., 2008, 2013; Strand *et al*., 2014). Furthermore, hypermethylation of circulating tumor DNA (ctDNA), which is shed from tumors into the bloodstream as a result of apoptosis, necrosis, and/or active secretion, can be detected in liquid biopsies (plasma/serum) from patients with PC (Polivka *et al*., 2015), suggesting a promising potential for development of noninvasive or minimally invasive diagnostic tests. So far, only a few candidate promoter methylation markers for PC have been analyzed in liquid biopsies, including genes known to be frequently hypermethylated in PC tissue samples, such as *GSTP1*,* APC*,* RAR2*, and *CDKN2A* (He and Bishop, 2016). However, whereas the specificity for PC of these individual candidate markers may be high in liquid biopsies, sensitivity is often suboptimal for diagnostic tests (He and Bishop, 2016; Yin *et al*., 2016).

Based on comprehensive epigenetic analyses of benign and malignant prostate tissue samples from multiple independent patient cohorts, we have previously identified and validated *CCDC181* (*C1orf114*) and *HAPLN3* as novel diagnostic and/or prognostic promoter hypermethylation candidate biomarkers for PC (Haldrup *et al*., 2013). Our previous study also showed that the promoter‐associated CpG islands of the two genes ST6(alpha‐N‐acetyl‐neuraminyl‐2,3‐beta‐galactosyl‐1,3)‐N‐acetylgalactosaminide alpha‐2,6‐sialyltransferase 3 (*ST6GALNAC3*) and zinc finger protein 660 (*ZNF660*) were significantly hypermethylated in PC versus benign prostate tissue in two small sample sets (Haldrup *et al*., 2013). However, further validation is needed to assess their diagnostic/prognostic biomarker potential for PC.

Accordingly, we have here conducted the first large‐scale study of *ST6GALNAC3* and *ZNF660* promoter methylation in PC. We report significant aberrant *ST6GALNAC3* and *ZNF660* promoter hypermethylation in PC in multiple large patient cohorts, as well as a significant association of *ZNF660* hypermethylation with high risk of BCR and reduced overall survival (OS) and prostate cancer‐specific survival (CSS). Furthermore, we provide the first proof‐of‐principle results, demonstrating that analysis of ctDNA methylation of *ST6GALNAC3*,* ZNF660*,* CCDC181*, and *HAPLN3* in liquid biopsies can identify PC patients with high specificity, thus warranting further investigations.

## 2 Materials and methods

### 2.1 Clinical samples used for quantitative methylation‐specific PCR

Radical prostatectomy cohort 1 consisted of 234 consecutive curatively intended RPs of histologically verified, clinically localized PC from patients treated at Department of Urology, Aarhus University Hospital, from 1997 to 2005 and was used for DNA methylation analysis by quantitative methylation‐specific PCR (qMSP), as previously described (Haldrup *et al*., 2013; Heeboll *et al*., 2009). For each patient, formalin‐fixed and paraffin‐embedded (FFPE) tissue was evaluated by a trained pathologist and regions with >90% tumor were marked on hematoxylin‐and‐eosin (HE)‐stained sections. Punch biopsies were obtained from the corresponding FFPE blocks. In total, 65 patients were excluded because of postoperative endocrine treatment (*n *=* *5) or poor DNA quality (*n *=* *60), leaving 169 RP patients for the data analysis (Table 1). For survival analyses, 11 additional patients who experienced BCR <3 months after RP were excluded. In addition, 20 adjacent normal (AN) and 10 prostatic intraepithelial neoplasia (PIN) samples from FFPE RP specimens, as well as 13 BPH samples, 15 primary tumor samples from patients with metastatic PC (MPC), and seven primary tumor samples from patients with castration‐resistant PC (CRPC) from FFPE transurethral resections of the prostate (TURP) specimens, were included. Of these, four AN, one PIN, and one BPH sample were excluded from the final data analysis because of poor DNA quality (Table 1).

**Table 1:** Clinicopathological characteristics of patients used for qMSP analyses of *ST6GALNAC3* and *ZNF660* promoter regions

PC samples	PC (*n *=* *169)	MPC (*n *=* *15)	CRPC (*n *=* *7)
Age, years			
Median (range)	62 (48–72)	79 (52–89)	63 (49–77)
Pathological Gleason score			
<7, *n* (%)	56 (33.1)	3 (20)	0 (0.0)
7, *n* (%)	77 (45.6)		
>7, *n* (%)	36 (21.3)	12 (80)	7 (100)
Pathological T‐stage			
≤pT2c, *n* (%)	115 (68.0)	NA	NA
≥pT3a, *n* (%)	54 (32.0)	NA	NA
Preoperative PSA			
<10 ng·mL^−1^, *n* (%)	44 (26.0)	1 (6.7)	1 (14.3)
≥10 ng·mL^−1^, *n* (%)	125 (74.0)	14 (93.3)	6 (85.7)
Nodal status			
pN0, *n* (%)	149 (88.2)	0 (0.0)	0 (0.0)
pN1, *n* (%)	0 (0.0)	1 (6.7)	2 (28.6)
Unknown, *n* (%)	20 (11.8)	14 (93.3)	5 (71.4)
Surgical margin status			
Negative, *n* (%)	112 (66.3)	NA	NA
Positive, *n* (%)	55 (32.5)	NA	NA
Unknown, *n* (%)	2 (1.2)	NA	NA
Biochemical recurrence status 36 months after radical prostatectomy			
No recurrence, *n* (%)	100 (59.2)	NA	NA
Recurrence, *n* (%)	59 (34.9)	NA	NA
Unknown (follow‐up <36 months)	10 (5.9)	NA	NA
Follow‐up, months			
Median (range)	124 (12–184)	NA	NA

Benign samples	BPH (*n *=* *12)	AN (*n *=* *16)	PIN (*n *=* *9)
Median age in years (range)	78 (65–92)	61 (56–72)	63 (55–68)

### 2.2 Quantitative methylation‐specific PCR

DNA was extracted from FFPE tissue samples using gDNA Eliminator columns from the miRNeasy FFPE Kit (Qiagen, Hilden, Germany) and bisulfite‐converted using the EZ‐96 DNA Methylation‐Gold™ Kit from Zymo Research (Irvine, CA, USA), as previously described (Haldrup *et al*., 2013). *ST6GALNAC3* and *ZNF660* qMSP primers and probes were designed using Primer3 (Untergasser *et al*., 2012) and positioned to target PC‐specific hypermethylated promoter regions, identified in previous bisulfite sequencing experiments (*ST6GALNAC3*: chromosome 1, 76540494‐76540569; *ZNF660*: chromosome 3, 44626539‐44626633, Hg19; Table S1) (Haldrup *et al*., 2013). For normalization and quality control, assays targeting CpG‐free genomic regions of *ALUC4* (Weisenberger *et al*., 2005) and *MYOD1* (Haldrup *et al*., 2013), respectively, were run in parallel. The relative methylation level of each candidate gene was determined by the candidate gene/*ALUC4* ratio. Samples with C_T(*MYOD1*)_>36 in at least 2/3 reactions were excluded from further analysis. For all reactions, 3 pmol of each primer, 1 pmol probe, 5 ng bisulfite‐converted template DNA, and TaqMan^®^ Universal PCR Master Mix No UNG (Applied Biosystems, Foster City, CA, USA) were used. Reactions (5 μL) were analyzed in triplicate in 384‐well plates using the 7900HT Fast Real‐Time PCR System (Applied Biosystems). For *ST6GALNAC3* and *MYOD1*, a PCR program of 50 °C for 2 min, 95 °C for 10 min, 40 cycles of 95 °C for 15 s, and 56 °C for 1 min was used. For *ZNF660* and *ALUC4*, annealing temperatures were adjusted to 60 °C and 58 °C, respectively. SDS 2.4 (Applied Biosystems) software was used to analyze all qMSP data.

### 2.3 Extraction of circulating cell‐free DNA from liquid biopsies

Serum samples from 10 patients with BPH and 27 patients with PC, collected at Department of Urology, Aarhus University Hospital, between 2004 and 2014, were used for methylation analysis of cfDNA (Table S2). Blood samples were collected prior to TURP or RP. Serum was isolated and stored at ‐80 °C within 3 h after blood draw. For cfDNA extraction, serum samples were thawed on ice. Samples <2 mL were supplemented with PBS to a total volume of 2 mL, while samples with volumes between 2 and 4 mL were supplemented with PBS to a total volume of 4 mL. A 182‐bp fragment of the soybean gene for cysteine‐rich polycomb‐like protein (CPP1) (Pallisgaard *et al*., 2013; Reinert *et al*., 2016) was spiked into each serum sample prior to cfDNA extraction, which was performed on the QIAsymphony robot (Qiagen) using the QIAsymphony Circulating DNA kit (192) (Qiagen). CfDNA was eluted in 60 μL WSE2 (Qiagen) elution buffer.

DNA extraction efficiencies were calculated from droplet digital PCR (ddPCR) analysis of the CPP1 spike‐in (Pallisgaard *et al*., 2013) and ranged from 32% to 62% (Table S2), as also previously reported (Pan *et al*., 2013). All serum samples were negative for lymphocyte DNA contamination, as assessed using a ddPCR assay (*PBC*) targeting immunoglobulin heavy‐chain rearrangements in B cells (Pallisgaard *et al*., 2013). The cfDNA concentration (haploid genome equivalents per mL) was estimated using another ddPCR assay (*Chr3*) targeting a gene‐free region on chromosome 3 (Reinert *et al*., 2016). For ddMSP, 2 × 20 μL extracted cfDNA was bisulfite‐converted in two reactions using the EZ DNA Methylation Direct™ kit (Zymo Research). For each patient, bisulfite‐converted cfDNA was eluted in 2 × 32 μL elution buffer and pooled for downstream analyses.

### 2.4 Droplet digital PCR

*Chr3*,* PBC*, and *CPP1* were analyzed by standard ddPCR, whereas *ST6GALNAC3*,* ZNF660*,* CCDC181*, and *HAPLN3* cfDNA promoter methylation was analyzed by ddMSP (Table S1).

For ddPCR analysis of *Chr3*,* PBC*, and *CPP1*, respectively, 11 μL ddPCR Supermix for Probes (Bio‐Rad, Hercules, CA, USA), 1 μL unconverted cfDNA extracted from serum, 18 pmol forward primer, 18 pmol reverse primer, 5 pmol probe, and nuclease‐free water were mixed to a total volume of 22 μL. Similar reaction conditions were used for the ddMSP assays (*ST6GALNAC3*,* ZNF660*,* CCDC181*, and *HAPLN3*), except that 9 μL bisulfite‐converted cfDNA was used as template. For all ddPCR and ddMSP experiments, droplets were generated on the automated droplet generator QX100 AutoDG™ (Bio‐Rad). ddPCR conditions for *CPP1*,* PBC*, and *Chr3* were 95 °C for 10 min, 45 cycles of 95 °C for 30 s, and 58 °C for 1 min. Similar PCR conditions were used for ddMSP, except that the annealing temperature was adjusted to 56 °C for *ST6GALNAC3* and to 60 °C for *ZNF660*,* CCDC181*, and *HAPLN3*. Droplets were read on the QX200™ Droplet Reader (Bio‐Rad), and samples were considered positive for methylation if ≥2 droplets were positive.

### 2.5 Statistical analysis

stata version 10.1 (StataCorp, College Station, TX, USA) was used for all statistical analyses. *P*‐values <0.05 were considered statistically significant. For statistical analyses, patients were dichotomized into high‐ and low‐methylation subgroups, using the median methylation level of *ST6GALNAC3* and *ZNF660*, respectively. Methylation differences were assessed using Mann–Whitney *U*‐tests. A nonparametric test was used to test for trends in methylation levels across ordered groups (BPH vs pT2 vs pT3‐4) (Cuzick, 1985). When relevant, *P*‐values were corrected for multiple testing using the Bonferroni method. Associations between *ST6GALNAC3* and *ZNF660* promoter methylation, clinicopathological variables, and transcriptional expression levels were assessed using Spearman's rank correlation coefficients or Mann–Whitney *U*‐tests. The diagnostic potential of candidate methylation markers was evaluated by ROC curve analysis.

For survival analysis using postoperative BCR (cutoff ≥0.2 ng·mL^−1^) as endpoint, patients not having experienced BCR were censored at their last normal PSA measurement. For OS and CSS analyses, surviving patients were censored at their last PSA measurement. Continuous variables were analyzed by uni‐ and multivariate Cox regression analyses, and dichotomized variables were analyzed by Kaplan–Meier analyses and log‐rank tests, as well as by uni‐ and multivariate Cox regression analyses.

### 2.6 450K DNA methylation array analysis

*ST6GALNAC3* and *ZNF660* promoter methylation levels were evaluated in two patient sample sets analyzed with the Illumina 450K BeadChip DNA methylation array (450K array).

Sample set 1 consisted of 20 PC (from RPs) and 21 NM (12 AN from RPs and 9 histologically normal from cystoprostatectomies) macrodissected fresh‐frozen samples (for clinicopathological data, see Table S3), as described previously (Haldrup *et al*., 2016; Moller *et al*., 2017; Strand *et al*., 2016). DNA from all samples was analyzed for DNA methylation using the 450K array by The Genome Centre Barts and the London School of Medicine and Dentistry, London, UK, as described previously (Strand *et al*., 2016).

Sample set 2 consisted of 11 AN and 19 PC fresh‐frozen laser microdissected RP samples (Table S3). Briefly, the samples were laser microdissected using the Veritas™ 704 (Arcturus) system from Applied Biosystems, and the extracted DNA was analyzed on the 450K array by AROS Applied Biotechnology A/S, as described previously (Haldrup *et al*., 2016).

### 2.7 Public RNAseq and 450K array data sets

RNAseq data for 52 AN and 495 PC tissue samples as well as 450K methylation array data for 50 AN and 497 PC tissue samples were downloaded from The Cancer Genome Atlas (TCGA). Of these, 494 PC and 35 AN samples had matched RNAseq and 450K array data. All samples with DNA methylation or RNA expression data available were used for AN vs. PC comparisons. For analysis of time to BCR, only PC patients with DNA methylation data available, as well as with ≥3 months of follow‐up, and no BCR <3 months after RP, were included (*n *=* *392; Table S3).

### 2.8 Data analysis, DNA methylation array, and RNAseq data

450K array methylation levels were reported as β‐values ranging from 0 (unmethylated) to 1 (completely methylated). Δβ was calculated for individual CpG sites as the median β‐value for PC samples minus the median β‐value for benign samples. No probes on the 450K array overlapped with the exact CpG sites targeted by the *ST6GALNAC3* and *ZNF660* qMSP assays; however, 8/8 and 4/5 CpG sites interrogated by probes on the 450K array in the promoter‐associated CpG islands of *ST6GALNAC3* and *ZNF660*, respectively, were significantly hypermethylated in PC compared to benign samples in each of the three patient tissue sample sets (*P *<* *0.05; Tables S4 and S5). Notably, the CpG site with the largest difference in methylation levels (Δβ) between PC and benign samples for each candidate gene was consistently the same in each of the three analyzed patient sample sets and was therefore considered to be representative for the gene and used for diagnostic and prognostic analyses. The representative 450K probe/CpG sites for *ST6GALNAC3* (cg21526205) and *ZNF660* (cg22598028) were located 5 base pairs downstream and 47 base pairs upstream of the corresponding qMSP assays, respectively.

For RNAseq data, reads were mapped to the genome using the Tuxedo Suite (Trapnell *et al*., 2012), and counts were calculated using HTSeq (Anders *et al*., 2015).

### 2.9 Ethical approval

All patient samples were collected at Department of Urology, Aarhus University Hospital, DK, from 1997 to 2014 with informed consent from all patients and approval from the relevant scientific ethical committees and the Danish Data Protection Agency.

## 3 Results

### 3.1 *ST6GALNAC3* and *ZNF660* promoter hypermethylation in prostate cancer tissue samples

**Figure 1:**
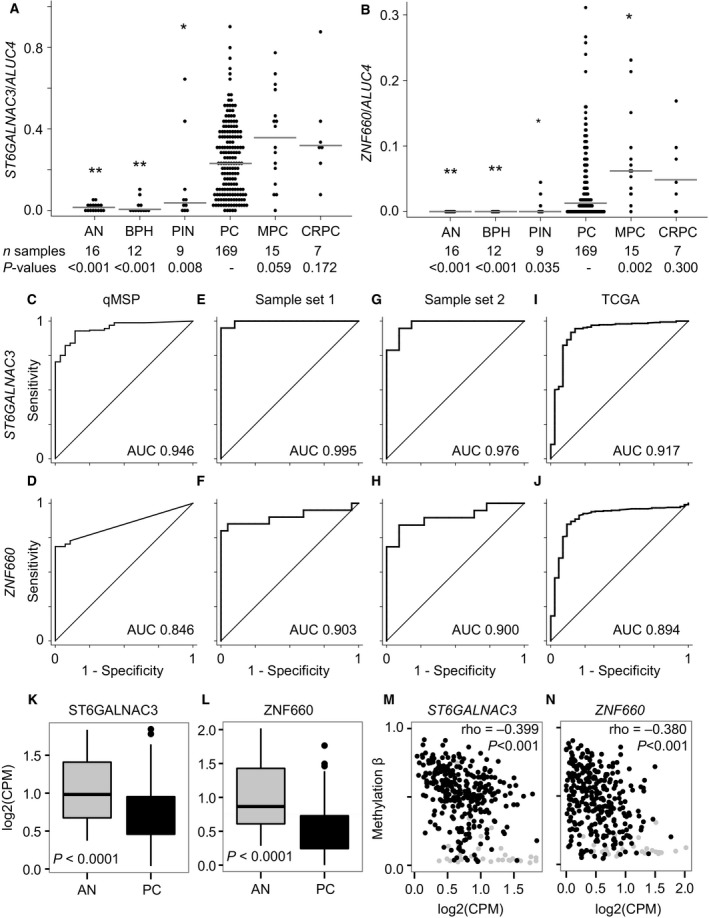
Promoter methylation and RNA expression of *ST6GALNAC3* and *ZNF660* in prostate tissue samples. (A, B) *ST6GALNAC3* and *ZNF660* qMSP data for samples of adjacent normal (AN), benign prostatic hyperplasia (BPH), prostatic intraepithelial neoplasia (PIN), localized prostate cancer from radical prostatectomies (PC, RP cohort 1), primary tumors from metastatic PC (MPC), and primary tumors from castrate‐resistant PC (CRPC). **P <* *0.001, *P <* *0.05 compared to PC samples. Gray lines: median methylation. *P*‐values from Mann–Whitney *U*‐tests. (C–J) Receiver operating characteristic (ROC) analysis comparing promoter methylation in nonmalignant and malignant tissue samples assayed by qMSP (C–D) or Illumina 450K DNA methylation array (E–J). 450K probes cg21526205 and cg22598028 for *ST6GALNAC3* and *ZNF660*, respectively, are shown. (C, D) 28 benign (16 AN and 12 BPH) versus 169 RP samples. (E, F) 11 AN versus 19 PC. (G, H) 21 benign (9 N and 12 AN) versus 20 PC. (I, J) 50 AN versus 497 PC. (K, L) mRNA expression in the TCGA cohort, 52 AN and 495 PC. (M, N) Correlation between promoter methylation and mRNA expression. In TCGA samples, 35 AN (gray dots) and 494 PC (black dots), Spearman correlation rho and *P*‐values are given.

To conduct a large‐scale evaluation of diagnostic and prognostic biomarker potential, we investigated *ST6GALNAC3* and *ZNF660* promoter methylation levels in 169 clinically localized PC samples (RP cohort 1) as well as in 16 AN, 12 BPH, 9 PIN, 15 MPC, and 7 CRPC tissue samples using qMSP (Table 1). For both *ST6GALNAC3* and *ZNF660,* RP samples were significantly hypermethylated compared to AN, BPH, and PIN samples (Fig. 1A,B; *P *<* *0.05), thus confirming and expanding on our previous findings (Haldrup *et al*., 2013). The median methylation level of *ST6GALNAC3* and *ZNF660* seemed to be further elevated in advanced PC samples (MPC and CRPC; Fig. 1A,B), although this was statistically significant only for *ZNF660* in MPC samples (Fig. 1B; *P *=* *0.002). Promoter methylation levels of both genes were similar in AN and BPH samples (Fig. 1A,B; *P *>* *0.1), and ROC curve analysis showed high discrimination between benign (AN and BPH) and PC samples with AUCs of 0.946 and 0.846 for *ST6GALNAC3* and *ZNF660*, respectively (Fig. 1C,D). At a fixed specificity of 100%, the sensitivity of *ST6GALNAC3* and *ZNF660* hypermethylation for PC was 70.4% and 68.6%, respectively.

For further large‐scale validation, we used 450K array data to investigate *ST6GALNAC3* and *ZNF660* promoter methylation levels in two independent patient sample sets (sample set 1: 21 NM vs 20 PC; sample set 2: 11 AN vs 19 PC) as well as in the publicly available TCGA RP cohort (50 AN vs 497 PC) (Table S3) (Cancer Genome Atlas Research, 2015). In ROC curve analysis, both *ST6GALNAC3* and *ZNF660* hypermethylation was highly cancer‐specific with AUCs ranging from 0.917 to 0.995 (*ST6GALNAC3*) and from 0.894 to 0.903 (*ZNF660*) in the three patient sets (Fig. 1E–J). Of note, *ST6GALNAC3* had the highest AUC in all four patient sample sets.

### 3.2 Promoter methylation and mRNA expression

To assess whether aberrant promoter hypermethylation of *ST6GALNAC3* and *ZNF660* in PC was associated with deregulation of transcriptional expression, we analyzed matched DNA methylation array and RNAseq data from the large TCGA patient cohort. ST6GALNAC3 and ZNF660 mRNA levels were significantly downregulated in PC compared to AN tissue samples (*P *<* *0.0001; Fig. 1K,L). Furthermore, the mRNA expression level of each gene was moderately, but significantly, inversely correlated with the promoter methylation level (*P *<* *0.001; Fig. 1M,N), collectively suggesting that aberrant promoter hypermethylation may contribute to downregulation of *ST6GALNAC3* and *ZNF660* in PC.

### 3.3 Correlation with clinicopathological variables

Next, to assess whether *ST6GALNAC3* and *ZNF660* promoter methylation levels in PC tissue samples could be associated with tumor aggressiveness, we evaluated possible associations between promoter methylation levels and standard clinicopathological prognostic variables. In RP cohort 1, the methylation level of *ZNF660* was significantly higher in Gleason score >7 vs. ≤7 tumors, and *ST6GALNAC3* methylation was significantly higher in PCs with positive vs. negative surgical margin status (Mann–Whitney *U*‐test, *P*‐value <0.05; Fig. S1). Correspondingly, in the TCGA RP cohort, high promoter methylation of both *ST6GALNAC3* and *ZNF660* was significantly associated with high pathological Gleason score (>7 vs. ≤7) in addition to high pathological T‐stage (>pT2 vs ≤pT2) and high preoperative PSA levels, and *ZNF660* hypermethylation was also associated with positive surgical margin status (Mann–Whitney *U*‐tests and Spearman correlations, *P *<* *0.05; Fig. S2). Thus, promoter hypermethylation of *ST6GALNAC3* and *ZNF660* was significantly associated with several adverse clinicopathological prognostic variables in two large independent RP cohorts.

### 3.4 Survival analysis

**Figure 2:**
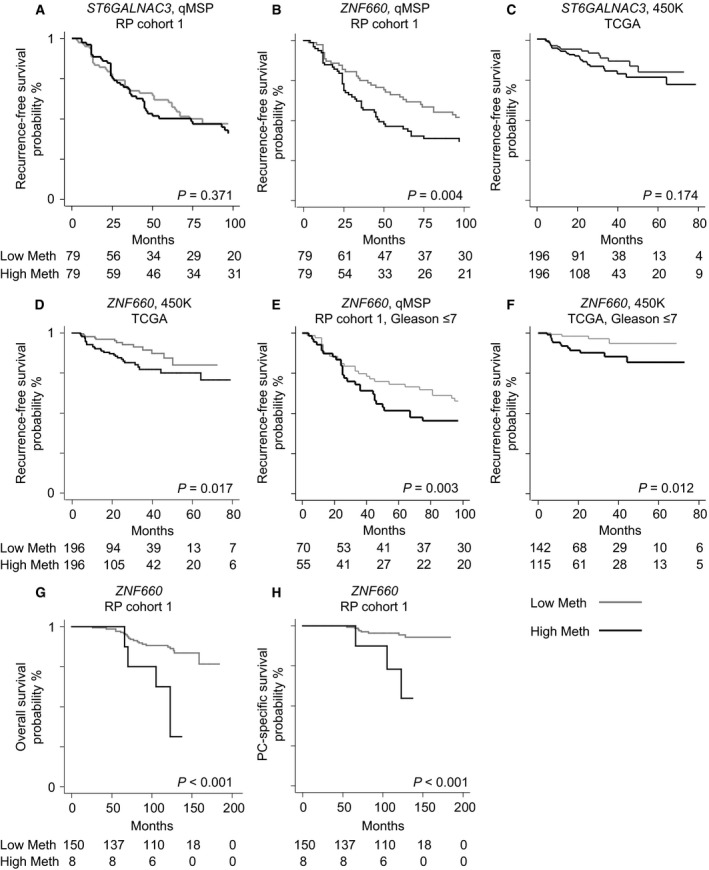
Kaplan–Meier plots. (A, B) *ST6GALNAC3* and *ZNF660* promoter methylation assayed by qMSP in RP cohort 1 divided into high and low methylation at the median; endpoint: biochemical recurrence after radical prostatectomy. (C, D) *ST6GALNAC3* and *ZNF660* promoter methylation assayed by 450K array in the TCGA cohort, samples divided into high and low methylation at the median; endpoint: biochemical recurrence after radical prostatectomy. (E, F) *ZNF660* promoter methylation in patients with Gleason score ≤7 in RP cohort 1 and the TCGA cohort. (G) *ZNF660* promoter methylation, dichotomized at top 5% most methylated; endpoint: overall survival. (H) *ZNF660* promoter methylation, dichotomized at top 5% most methylated; endpoint: PC‐specific survival. *P*‐values from log‐rank tests.

To further evaluate the prognostic potential of *ST6GALNAC3* and *ZNF660* promoter methylation, we performed survival analyses using BCR as endpoint. Patients in RP cohort 1 with promoter methylation levels above/below the median were classified into high‐/low‐methylation subgroups for *ST6GALNAC3* and *ZNF660*, respectively. While no significant associations were seen for *ST6GALNAC3* (Fig. 2A and Table 2), *ZNF660* promoter hypermethylation was significantly associated with BCR after RP in both univariate Cox regression (*P *=* *0.005; Table 2) and Kaplan–Meier analysis (*P *=* *0.004; Fig. 2B). At 48 months post‐RP, 54 vs. 31% of the patients in the high‐ and low‐*ZNF660* promoter methylation subgroups had experienced PSA recurrence, respectively. Similarly, in the external TCGA RP cohort (used for validation), *ST6GALNAC3* promoter methylation did not predict BCR (*P *>* *0.05; Table 2, Fig. 2C), whereas *ZNF660* hypermethylation was significantly associated with higher risk of BCR in both univariate Cox regression (*P *=* *0.02; Table 2) and Kaplan–Meier analysis (*P *=* *0.017; Fig. 2D). Although *ZNF660* was only borderline significant or nonsignificant after adjustment for routine clinicopathological factors in multivariate cox regression analysis (*P *=* *0.092/0.165; Table 2), Harrell's C‐index increased in both cohorts by the addition of *ZNF660* to a multivariate model based on clinicopathological variables only (Table 2), suggesting improved predictive accuracy.

**Table 2:** Cox regression analyses of time to biochemical recurrence after radical prostatectomy. CI, confidence interval. *ST6GALNAC3* and *ZNF660* methylation levels were dichotomized at the median methylation level for all patients

	Variables	Univariate HR (95% CI)	*P*	Multivariate HR (95% CI)	*P*	Harrell's C‐index incl. *ZNF660*	Harrell's C‐index excl. *ZNF660*
RP cohort 1	*ST6GALNAC3, low vs. high*	1.21 (0.792–1.852)	0.376	–	–		
	*ZNF660, low vs. high*	1.85 (1.201–2.857)	**0.005**	1.48 (0.938–2.337)	0.092	0.732	
	Gleason ≤7 vs >7	2.29 (1.390–3.784)	**<0.001**	1.78 (1.065–2.964)	**0.028**		0.726
	pT ≤2 vs ≥3	3.19 (2.064–4.919)	**<0.001**	2.01 (1.193–3.381)	**0.009**		
	Margin neg. vs pos.	3.44 (2.224–5.327)	**<0.001**	1.98 (1.171–3.347)	**0.011**		
	PSA (cont.)	1.04 (1.025–1.063)	**<0.001**	1.04 (1.021–1.060)	**<0.001**		
TCGA RP cohort	*ST6GALNAC3, low vs. high*	1.51 (0.830–2.750)	0.177	–	–		
	*ZNF660, low vs. high*	2.11 (1.127–3.962)	**0.020**	1.57 (0.830–2.960)	0.165	0.703	
	Gleason ≤7 vs >7	3.34 (1.834–6.076)	**<0.000**	2.35 (1.273–4.350)	**0.006**		0.675
	pT ≤2 vs ≥3	5.96 (2.137–16.653)	**0.001**	4.22 (1.474–12.086)	**0.007**		
	Margin neg. vs pos.	1.47 (0.814–2.642)	0.202	–	–		
	PSA (cont.)	1.02 (0.999–1.045)	0.062	–	–		

Significant *P*‐values are highlighted in bold.

In present clinical practice, risk stratification is particularly difficult for patients diagnosed with low‐ to intermediate‐grade PC (Gleason score ≤7). We therefore evaluated whether *ZNF660* promoter methylation may help determine PC aggressiveness in this patient subgroup. In RP cohort 1, *ZNF660* promoter hypermethylation was significantly associated with BCR in Cox regression analysis (hazard ratio (HR) 1.78, *P *=* *0.025; Table 3) and in Kaplan–Meier analysis (*P *=* *0.003; Fig. 2E). This was also validated in the TCGA cohort (Cox regression analysis, HR 3.79, *P *=* *0.020; Table 3; Kaplan–Meier analysis, *P *=* *0.012; Fig. 2F). In multivariate analyses, *ZNF660* promoter hypermethylation was borderline significant after adjustment for clinicopathological variables (*P *=* *0.106/0.054; Table 3), whereas Harrell's C‐index was improved in both cohorts by the addition of *ZNF660* to clinicopathological variables (Table 3).

**Table 3:** Cox regression analyses of time to biochemical recurrence after radical prostatectomy in patients with Gleason score ≤7. CI, confidence interval. *ST6GALNAC3* and *ZNF660* methylation levels were dichotomized at the median methylation level for all patients

	Variables	Univariate HR (95% CI)	*P*	Multivariate HR (95% CI)	*P*	Harrell's C‐index incl. *ZNF660*	Harrell's C‐index excl. *ZNF660*
RP cohort 1	*ZNF660, low vs. high*	1.78 (1.074–2.955)	**0.025**	1.53 (0.914–2.552)	0.106	0.732	
	pT ≤2 vs ≥3	3.59 (2.150–5.983)	**<0.001**	1.88 (1.002–3.526)	**0.049**		0.726
	Margin neg. vs pos.	4.11 (2.459–6.856)	**<0.001**	2.61 (1.405–4.845)	**0.002**		
	PSA (cont.)	1.05 (1.024–1.068)	**<0.001**	1.03 (1.011–1.057)	**0.004**		
TCGA RP cohort	*ZNF660, low vs. high*	3.79 (1.234–11.616)	**0.020**	3.03 (0.983–9.348)	0.054	0.727	
	pT ≤2 vs ≥3	6.24 (1.427–27.308)	**0.015**	5.31 (1.206–23.388)	**0.027**		0.650
	Margin neg. vs pos.	1.08 (0.381–3.080)	0.880	–	–		
	PSA (cont.)	1.00 (0.926–1.069)	0.891	–	–		

Significant *P*‐values are highlighted in bold.

For further assessment of prognostic potential, we used OS and CSS as endpoints for survival analysis in RP cohort 1, where long‐term clinical follow‐up data were available. Given the relatively low number of events (26 patients had died, including 11 PC‐specific deaths), we tested only the most highly methylated RP patients for associations with OS/CSS. The top 5% of the RP patients with the highest methylation level of *ZNF660* had significantly reduced OS compared to the rest of the patients in both univariate Cox regression and Kaplan–Meier analysis (Table 4, Fig. 2G; *P *<* *0.05). High *ZNF660* promoter methylation was also significantly associated with poor CCS in Cox regression and Kaplan–Meier analysis (Table 4, Fig. 2H; *P *<* *0.05).

**Table 4:** Univariate Cox regression analyses. For cox regression analysis of *ZNF660* promoter methylation, the 5% patients with the highest methylation level were compared to the 95% less methylated. CI, confidence interval; Cont., continuous; Dich., dichotomized

	Variables	Univariate HR (95% CI)	*P*
RP cohort 1	*ZNF660*, low vs. high	5.09 (1.900–13.626)	**0.001**
Overall survival	Gleason ≤7 vs >7	3.89 (1.798–8.428)	**0.001**
	pT ≤2 vs ≥3	1.11 (0.477–2.563)	0.814
	Margin pos. vs neg.	1.15 (0.513–2.585)	0.732
	PSA (cont.)	1.01 (0.976–1.047)	0.559
	Age at RP (cont.)	1.06 (0.976–1.140)	0.175
RP cohort 1	*ZNF660*, low vs. high	7.70 (2.020–29.327)	**0.003**
PC‐specific survival	Gleason ≤7 vs >7	7.91 (2.311–27.077)	**0.001**
	pT ≤2 vs ≥3	1.95 (0.596–6.395)	0.270
	Margin pos. vs neg.	1.24 (0.362–4.230)	0.734
	PSA (cont.)	1.03 (0.980–1.075)	0.267
	Age at RP (cont.)	1.03 (0.912–1.153)	0.674

Significant *P*‐values are highlighted in bold.

### 3.5 The potential of methylated ctDNA as novel PC biomarkers in liquid biopsies

Finally, as proof of principle, we investigated whether hypermethylated ctDNA for *ST6GALNAC3* and *ZNF660*, as well as for two of our previously identified top candidate hypermethylated genes in PC tissue (*CCDC181* and *HAPLN3* (Haldrup *et al*., 2013)), could be detected in serum samples from patients with PC.

**Figure 3:**
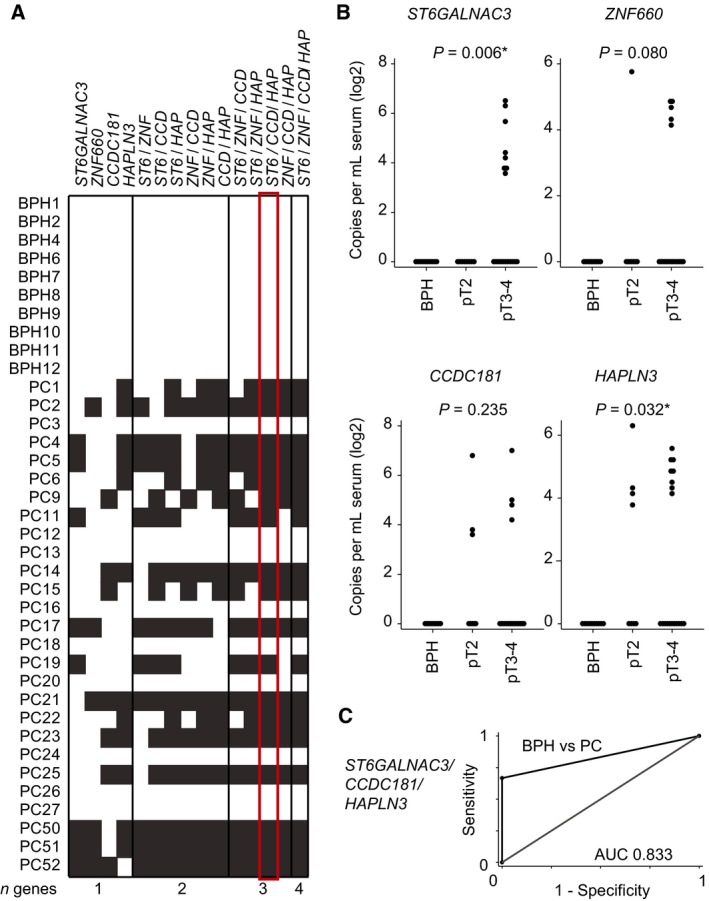
Promoter methylation in cfDNA from serum. (A) Heatmap of promoter methylation detected in cfDNA extracted from serum samples for single genes and combinations of genes. White: no methylation detected; gray: methylation detected; red box: best minimal combination of markers (highest AUC). (B) Copies per mL of hypermethylated *ST6GALNAC3*,*ZNF660*,*CCDC181*, and *HAPLN3* in serum samples from 10 patients with BPH and 27 patients with PC analyzed by droplet digital PCR. *P*‐values for trend of methylation in BPH<pT2 < pT3‐4 are given. (C) ROC curve analysis of the three‐gene panel *ST6GALNAC3*/*CCDC181*/*HAPLN3* comparing serum from patients with BPH to patients with PC. *P <* *0.05, test for trend BPH<pT2 < pT3‐4.

Thus, cfDNA from 27 patients with PC and 10 patients with BPH (Table S2) was analyzed by ddMSP. We detected hypermethylated ctDNA in 22% (*ZNF660*), 26% (*CCDC181*), 31% (*ST6GALNAC3*), and 44% (*HAPLN3*) of the patients with PC (Table 5 and Fig. 3A,B), whereas all serum samples from patients with BPH were negative for all four genes (i.e., corresponding to 100% specificity and 22–44% sensitivity for PC). The level of hypermethylated ctDNA detected in serum samples from patients with PC correlated positively with pathological T‐stage for *ST6GALNAC3* and *HAPLN3* (test for trend, *P *<* *0.05; Fig. 3B) and was borderline significant for *ZNF660* (*P *=* *0.08) but nonsignificant for *CCDC181* (*P *=* *0.235) (Fig. 3B). We found no significant associations between Gleason score, surgical margin status, or preoperative PSA levels and hypermethylated ctDNA levels for any of the genes in this patient sample set (data not shown).

**Table 5:** Serum samples positive for hypermethylated DNA fragments

	BPH	pT2	pT3‐4	All PC
*ST6GALNAC3*	0/10 (0%)	0/8 (0%)	8/19 (42%)	8/27 (30%)
*ZNF660*	0/10 (0%)	1/8 (13%)	5/19 (26%)	6/27 (22%)
*CCDC181*	0/10 (0%)	3/8 (38%)	4/19 (21%)	7/27 (26%)
*HAPLN3*	0/10 (0%)	4/8 (50%)	8/19 (42%)	12/27 (44%)

To evaluate whether multimarker panels could improve sensitivity over single ctDNA candidate biomarkers, we analyzed all possible combinations of *ST6GALNAC3*,* ZNF660*,* CCDC181*, and *HAPLN3* in serum (Fig. 3A; Table S6). The three‐gene panel *ST6GALNAC3*/*CCDC181/HAPLN3* was the best performing minimal multimarker panel in our patient set, showing 67% sensitivity and 100% specificity for patients with PC compared to patients with BPH and a corresponding AUC of 0.833 in ROC curve analysis (Fig. 3A (red box), Fig. 3C, and Table S6), thus clearly improving sensitivity over the best performing single marker (*HAPLN3*).

## 4 Discussion

In this large‐scale study, we identified *ST6GALNAC3* and *ZNF660* as highly frequent targets of aberrant promoter hypermethylation in PC after analysis of four independent patient sets, including a total of 110 NM and 705 PC tissue samples. Furthermore, *ZNF660* hypermethylation was significantly associated with postoperative BCR in two large independent RP cohorts, including in the subgroup of patients with Gleason score ≤7. Hypermethylation of *ZNF660* was also significantly associated with decreased OS and CSS after RP in a cohort with long clinical follow‐up time (HRs 5.09 and 7.70, respectively), together suggesting that *ZNF660* has the potential to improve risk stratification for patients with PC. In addition, hypermethylation of both genes in cfDNA extracted from serum, as well as of the additional candidate hypermethylation markers *CCDC181* and *HAPLN3*, was highly specific for patients with PC compared to patients with BPH. The combination *ST6GALNAC3*/*CCDC181*/*HAPLN3* in serum had a specificity of 100% and a sensitivity of 67% for PC. In summary, this is the first large‐scale study to demonstrate PC‐specific hypermethylation of *ST6GALNAC3* and *ZNF660* and prognostic value of *ZNF660* hypermethylation for BCR, OS, and CSS, as well as to demonstrate biomarker potential of *ST6GALNAC3*,* ZNF660*,* CCDC181*, and *HAPLN3* ctDNA hypermethylation for PC.

We here found cancer‐specific promoter hypermethylation of *ST6GALNAC3* and *ZNF660* in PC compared to benign prostate tissue samples in four different patient sets. The AUCs of *ST6GALNAC3* (0.917–0.995) were generally higher than those of *ZNF660* (0.846–0.903) and were similar to those of other published diagnostic candidate methylation markers (Goering *et al*., 2012; Haldrup *et al*., 2013, 2016; Kristensen *et al*., 2014), suggesting that *ST6GALNAC3* has promising diagnostic biomarker potential for PC tissue. To further assess this, it would be interesting to evaluate *ST6GALNAC3* and *ZNF660* promoter methylation also in cancer‐negative diagnostic biopsies, as promoter hypermethylation field effects in cancer‐negative diagnostic biopsies have previously been shown to predict the result of repeat biopsy (Moller *et al*., 2017; Partin *et al*., 2014; Stewart *et al*., 2013; Trock *et al*., 2012; Troyer *et al*., 2009; Van Neste *et al*., 2016). Results of such analyses should, for example, be compared to the commercially available urine‐based PCA3 assay (Progensa) and the tissue‐based methylation test ConfirmMDx (MDxHealth), both designed to determine whether a repeat biopsy is necessary. Notably, up to 25% of PC‐negative biopsies may be false negatives, and better biomarkers could reduce the number of unnecessary repeat biopsies (Bakardzhiev *et al*., 2012; Blute *et al*., 2015; Ploussard *et al*., 2013; Tan *et al*., 2008).

We also analyzed the association between *ZNF660* promoter hypermethylation and patient prognosis. In univariate analyses, *ZNF660* hypermethylation was significantly associated with early BCR in two RP cohorts. For the full cohort as well as the subgroup of patients with low‐ to intermediate‐grade PC, *ZNF660* improved Harrell's C‐index when combined with clinicopathological parameters, although it was only borderline significant or nonsignificant in multivariate Cox regression analyses (Tables 2 and 3). Although these results suggest that *ZNF660* has the potential to provide independent prognostic value beyond routine clinicopathological parameter, further large‐scale studies are needed to clarify this. Furthermore, a potential limitation of the present study is the use of RP specimens for prognostic assessment. Future studies should evaluate *ZNF660* promoter methylation levels in diagnostic biopsies to determine whether *ZNF660* alone or in combination with other molecular markers may improve risk stratification and thus guide treatment decisions. Importantly, only patients with aggressive PC should undergo RP or radiation therapy, while patients with nonaggressive PC are candidates for AS (Mottet *et al*., 2017). Moreover, as only preoperative clinicopathological parameters are available at the time of diagnosis, molecular markers are likely to contribute relatively more independent prognostic information at this point than after RP. Notably, the association of *ZNF660* promoter hypermethylation with OS and CSS supports a potential role of *ZNF660* hypermethylation as a biomarker for aggressive PC. Very few studies have previously shown an association between promoter hypermethylation and survival of patients with PC (Richiardi *et al*., 2009; Torres‐Ferreira *et al*., 2017); however, to solidly establish the association between *ZNF660* promoter methylation levels and PC aggressiveness, larger patient cohorts with longer follow‐up and more OS/CSS events should be analyzed.

This is the first study to show that hypermethylation of *ST6GALNAC3*,* ZNF660*,* CCDC181*, and *HAPLN3* can be detected in liquid biopsies from patients with PC. Methylated DNA has high potential as a biomarker, as it is frequently highly cancer‐specific, can be measured in, for example, tissue, blood, or urine, is easily detected with standard PCR‐based methods, and is relatively stable compared to RNA (Costa‐Pinheiro *et al*., 2015). The presence of hypermethylated ctDNA was 100% cancer‐specific in our patient set for all four candidate genes investigated. Whereas each individual gene had a relatively low sensitivity for PC (22–44%), combinations of *ST6GALNAC3*,* ZNF660*,* CCDC181*, and *HAPLN3* ctDNA methylation had 100% specificity and 37–67% sensitivity; thus, by combining multiple ctDNA markers, we increased the sensitivity while retaining 100% specificity. Previous studies investigating hypermethylated ctDNA in liquid biopsies (plasma/serum) from patients with PC reported similar specificities and sensitivities for single genes, for example, *EDNRB*,* GSTP1,* and *RASSF1a* (He and Bishop, 2016; Wu *et al*., 2011). Few studies have attempted to combine multiple ctDNA hypermethylation markers for PC, and, in contrast to our results, these reported only a minor increase in sensitivity (Ellinger *et al*., 2008; Sunami *et al*., 2009). However, in the earlier studies, the sensitivity of the majority of the included markers was relatively low (1–13%), possibly explaining the limited benefit of combining multiple candidate markers in these reports.

Minimally invasive ctDNA‐based tests for PC in liquid biopsies could add specificity to PC detection as compared to the currently used serum PSA test, which has suboptimal positive predictive value (Mistry and Cable, 2003; Salman *et al*., 2015), and thus also could possibly help to reduce the number of unnecessary prostate biopsies. Recently, the Stockholm‐3 model (STLHM3), the 4‐Kallikrein (4Kscore), and the Prostate Health Index (phi) test have all been suggested as alternatives to PSA for detection of clinically significant PC by subsequent prostate biopsy (Braun *et al*., 2016; Eklund *et al*., 2016; Loeb *et al*., 2015). Hypermethylation of ctDNA is not included in any of the tests; however, given the high specificity shown by us and others (He and Bishop, 2016), it is possible that addition of ctDNA hypermethylation markers could improve such tests. The present proof‐of‐concept study was based on a relatively small patient sample set, and larger‐scale clinical studies are needed to assess the actual diagnostic potential of hypermethylated *ST6GALNAC3*,* ZNF660*,* CCDC181*, and *HAPLN3* in serum, as well as in other biofluids such as plasma and urine.

Here, we found a significant correlation between hypermethylated *ST6GALNAC3* and *HAPLN3* ctDNA levels in serum and higher pathological T‐stage. Similar associations have also been reported in previous studies which suggested that *RASSF1*,* RARB2*, and/or *GSTP1* ctDNA methylation may predict the stage and grade of localized PC (Sunami *et al*., 2009). Furthermore, it has been proposed that *GSTP1*,* SRD5A2*,* CYPIIAI*, and *PCDH17* ctDNA methylation may predict BCR risk after RP (Bastian *et al*., 2005; Horning *et al*., 2015; Lin *et al*., 2015). Conceivably, the ctDNA methylation level in a liquid biopsy may therefore help assess PC aggressiveness at diagnosis. However, given the small number of samples in the present analysis, larger studies are needed to examine the association between PC aggressiveness and ctDNA promoter hypermethylation of *ST6GALNAC3*,* ZNF660*,* CCDC181*, and *HAPLN3*, possibly in combination with the previously identified ctDNA methylation markers suggested to be associated with PC aggressiveness.

In the current study, we also found that *ST6GALNAC3* and *ZNF660* expression was significantly downregulated in PC tissue samples, suggesting that aberrant promoter hypermethylation and transcriptional silencing of these genes might play a role in PC development and/or progression. *ST6GALNAC3* catalyzes the transfer of sialic acid onto GalNAc and is member of a family of sialyltransferases involved in generating a large number of differently sialylated glycoproteins and lipids (Pearce and Laubli, 2016; Tsuchida *et al*., 2005). Whereas other sialyltransferases have been reported to be upregulated in many cancer types, including PC (Hatano *et al*., 2011; Pearce and Laubli, 2016), *ST6GALNAC3* has only previously been investigated in renal cancer, where transcriptional expression was found to be downregulated in cancer cell lines but unaltered in clinical cancer tissue samples (Pearce and Laubli, 2016; Senda *et al*., 2007). The sialic acid level seems to be generally increased on the cell surface of cancer cells and is thought to play a central role in cancer biology and cancer immunity in many cancers, including PC (Pearce and Laubli, 2016). The potential biological role of *ST6GALNAC3* downregulation in PC, as observed in the present study, is unclear. It is, however, possible that *ST6GALNAC3* expression levels could affect the composition of sialic acids on the cell surface. *ZNF660* is a member of the zinc finger family of proteins and is likely a transcription factor. Whereas other zinc finger proteins have been associated with, for example, cancer progression in multiple cancer types, including PC (Jen and Wang, 2016), the role of *ZNF660* in cancer has not been investigated. Thus, the function of both *ST6GALNAC3* and *ZNF660* in benign and malignant prostate cells should be investigated in future studies.

In conclusion, we here conducted a large‐scale study showing highly cancer‐specific *ST6GALNAC3* and *ZNF660* hypermethylation, demonstrating promising diagnostic potential. We furthermore identify *ZNF660* hypermethylation as associated with increased risk of BCR, as well as with reduced OS and reduced CSS, thus showing promising prognostic potential. Also, as proof of principle, we demonstrate hypermethylation of four genes in liquid biopsies from patients with PC and identify a three‐gene combination with 100% specificity and 67% sensitivity for PC, suggesting a high potential as novel minimally invasive ctDNA methylation markers for PC.

## Author contributions

CH and KDS conceived and designed the project. CH, TFØ, and KDS supervised the project. ALP conducted all qMSP analyses and NØ performed all ddMSP analyses. SHS contributed 450K data for sample set 1. CH contributed 450K data for sample set 2. CH analyzed data, including all statistical analyses, and prepared all figures and tables. SH and MB provided tissue and serum samples from patients with BPH and patients with PC, as well as clinicopathological data. CH and KDS wrote the manuscript. All authors critically revised and approved the manuscript.

## Conflict of interest

Karina D. Sørensen has received consultancy fees from Exiqon A/S. Christa Haldrup, Torben F. Ørntoft, and Karina D. Sørensen are co‐inventors on a patent application (Application No./Patent No.: 13732819.1‐1403) regarding *CCDC181* (*C1orf114*), *HAPLN3*,* ST6GALNAC3*, and *ZNF660* hypermethylation as biomarkers for prostate cancer.

## Supporting information


**Table S1**. Sequences of primers and probes used for qMSP, ddPCR and ddMSP.
**Table S2**. Serum samples used for DNA extraction.
**Table S3.** Clinicopathological data for patient sample sets analyzed on the 450K array for DNA methylation.
**Table S4.** *ST6GALNAC3* Illumina 450K DNA methylation array data for Sample set 1: 20 PC and 21 benign (9 N and 12 AN), Sample set 2: 19 PC and 11 AN, and TCGA: 497 PC and 50 AN.
**Table S5.** *ZNF660* Illumina 450K DNA methylation array data for Sample set 1: 20 PC and 21 benign (9 N and 12 AN), Sample set 2: 19 PC and 11 AN, and TCGA: 497 PC and 50 AN.
**Table S6.** AUCs, sensitivities and specificities of methylation versus no methylation in serum samples.
**Fig. S1.** Correlation of promoter methylation, as determined by qMSP, to clinicopathological parameters in RP cohort 1 (*n *=* *169).
**Fig. S2.** Correlation of promoter methylation, as determined by 450K array, to clinicopathological parameters in the TCGA RP cohort (*n *=* *497).
