# Supplementary material for: Biomarker potential of ST6GALNAC3 and ZNF660 promoter hypermethylation in prostate cancer tissue and liquid biopsies
Source: Mol Oncol. 2018 Mar 13;12(4):545–60. doi: 10.1002/1878-0261.12183 (PMC5891052; doi:10.1002/1878-0261.12183)
Supplement: Supplementary file 1 — Table S1. Sequences of primers and probes used for qMSP, ddPCR and ddMSP. Table S2. Serum samples used for DNA extraction. Table S3. Clinicopathological data for patient sample sets analyzed on the 450K array for DNA methylation. Table S4. ST6GALNAC3 Illumina 450K DNA methylation array data for Sample set 1: 20 PC and 21 benign (9 N and 12 AN), Sample set 2: 19 PC and 11 AN, and TCGA: 497 PC and 50 AN. Table S5. ZNF660 Illumina 450K DNA methylation array data for Sample set 1: 20 PC and 21 benign (9 N and 12 AN), Sample set 2: 19 PC and 11 AN, and TCGA: 497 PC and 50 AN. Table S6. AUCs, sensitivities and specificities of methylation versus no methylation in serum samples. Fig. S1. Correlation of promoter methylation, as determined by qMSP, to clinicopathological parameters in RP cohort 1 (n = 169). Fig. S2. Correlation of promoter methylation, as determined by 450K array, to clinicopathological parameters in the TCGA RP cohort (n = 497). [file MOL2-12-545-s001.docx]

Supporting Information for: “Biomarker potential of *ST6GALNAC3* and *ZNF660* promoter hypermethylation in prostate cancer tissue and liquid biopsies’ by Haldrup *et al.*, Molecular Oncology 2018

Contents:

Supplementary Tables 1-6

Supplementary Figures 1-2

References

| Gene | Forward primer 5’-3’ | Reverse primer 5’-3’ | Probe 5’-3’ | Application |
| --- | --- | --- | --- | --- |
| *ST6GALNAC3* | GATTGTTTTTGATTTAGGCG | CTTCAAAATACAAACCATAACG | FAM-GTTCGGTGGTAGGAGGGTCG-BHQ1 | qMSP and ddMSP |
| *ZNF660* | GGGTTTTGGGAGCGTGT | AACGACCCCTTAACTACCG | FAM-TTGCGGTTGTTCGTTGTTGAGAT-BHQ1 | qMSP and ddMSP |
| *ALUC4^1^* | GGTTAGGTATAGTGGTTTATATTTGTAATTTTAGTA | ATTAACTAAACTAATCTTAAACTCCTAAC | FAM-CCTACCTTAACCTCCC-MGBNFQ | qMSP |
| *MYOD1^2^* | CCAACTCCAAATCCCCTCTCTAT | TGGTTTTTTTTAGGGAGTAAGTTTGTT | FAM-TCCCTTCCTATTCCTAAATCCAACCTAAATACCTCC-BHQ1 | qMSP |
| *CCDC181^2^* | ATTTGCGTAGGCGTATGTAA | CGTAAATTCCTTAATCGTCCC | HEX-  TCGGTGTTTGCGAAGGGTTAG-BHQ1 | ddMSP |
| *HAPLN3^2^* | CTTTTCGTAGTGTTCGGTTTAC | GAATTCCTCCCTTACCGC | HEX-TCGGATTTTGTTCGGGAGGT-BHQ1 | ddMSP |
| *Chr3^3^* | CTAGAAGATCTACCTCCAAGAGG | CCAGGCTGAAGCTATTCCAG | FAM-CTCATACATCTGGCATATGGGCTGG-BHQ1 | ddPCR |

Supplementary table 1: Sequences of primers and probes used for qMSP, ddPCR and ddMSP.

^1^ Assay from Weisenberger et al. ([Weisenberger et al., 2005](#_ENREF_3))

^2^ Assay from Haldrup et al. ([Haldrup et al., 2013](#_ENREF_1))

^3^ Assay from Reinert et al. ([Reinert et al., 2016](#_ENREF_2))

| Patient | Path. T-stage | Path. Gleason score | PSA | Age | Serum (mL) | Extraction efficiency* (%) | Serum total Copies (chr3) | Serum copies per mL (chr3) |
| --- | --- | --- | --- | --- | --- | --- | --- | --- |
| BPH1 | - | - | 1.7 | 65 | 1.3 | 46.1 | 57915 | 44550 |
| BPH2 | - | - | 1.9 | 61 | 1.7 | 40.5 | 83160 | 48918 |
| BPH4 | - | - | 1.2 | 71 | 3.3 | 58. 7 | 43560 | 13200 |
| BPH6 | - | - | 3.9 | 68 | 2.8 | 39.5 | 36383 | 12994 |
| BPH7 | - | - | 4.3 | 63 | 3.4 | 52.3 | 57173 | 16815 |
| BPH8 | - | - | 0.6 | 71 | 1.9 | 45.3 | 71280 | 37516 |
| BPH9 | - | - | 3.8 | 61 | 3.2 | 55.2 | 37125 | 11602 |
| BPH10 | - | - | 2.4 | 66 | 2.1 | 62.4 | 132165 | 62936 |
| BPH11 | - | - | 0.6 | 70 | 2.9 | 54.4 | 73508 | 25347 |
| BPH12 | - | - | 2.8 | 66 | 2.6 | 41.9 | 21533 | 8444 |
| PC1 | T3b | 6 | 13.3 | 68 | 1.3 | 44.8 | 17820 | 13708 |
| PC2 | T3b | 8 | 41.3 | 75 | 2.2 | 41.1 | 33413 | 15188 |
| PC3 | T3B | 7 | 12.9 | 65 | 1.5 | 42.9 | 73508 | 49005 |
| PC4 | T3b | 5 | 5.6 | 67 | 1.5 | 34.1 | 49748 | 33165 |
| PC5 | T3b | 7 | 24 | 69 | 2.2 | 37.6 | 42323 | 19238 |
| PC6 | T3b | 7 | 64.5 | 67 | 1.4 | 42.4 | 12623 | 9016 |
| PC9 | T3c | 7 | 7.7 | 67 | 2.2 | 44.5 | 104693 | 47588 |
| PC11 | T4 | 8 | 17.5 | 65 | 2.2 | 38.9 | 18563 | 8438 |
| PC12 | T3c | 7 | 8 | 72 | 2.2 | 38.4 | 16335 | 7425 |
| PC13 | T3c | 7 | 27.7 | 63 | 2.2 | 58.9 | 36383 | 16538 |
| PC14 | T3c | 7 | 8.1 | 60 | 1.9 | 39.5 | 104693 | 55101 |
| PC15 | T3c | 7 | 10.8 | 67 | 1.6 | 32.3 | 17820 | 11497 |
| PC16 | T3c | 8 | 6.6 | 69 | 2.2 | 36.0 | 14850 | 6750 |
| PC17 | T3b | 7 | 18.6 | 72 | 2.2 | 44.3 | 21533 | 9788 |
| PC18 | T4 | 7 | 16.1 | 69 | 2.2 | 53.3 | 52718 | 23963 |
| PC19 | T4 | 7 | 21.3 | 63 | 2.2 | 41.3 | 25245 | 11475 |
| PC20 | T1c | 6 | 5.2 | 50 | 2.2 | 44.5 | 49748 | 22613 |
| PC21 | T2 | 6 | 17 | 66 | 2.2 | 53.9 | 35640 | 16200 |
| PC22 | T2a | 6 | 10.8 | 52 | 2.9 | 42.7 | 40095 | 13826 |
| PC23 | T2c | 6 | 5.7 | 55 | 3.2 | 49.1 | 93555 | 29236 |
| PC24 | T2c | 6 | 5.9 | 49 | 2.8 | 53.3 | 178943 | 63908 |
| PC25 | T2c | 7 | 5.7 | 65 | 3.3 | 52.3 | 56430 | 17100 |
| PC26 | T2c | 7 | 5.8 | 59 | 3.4 | 34.1 | 26730 | 7862 |
| PC27 | T2 | 6 | 5.1 | 56 | 3.3 | 41.6 | 122513 | 37125 |
| PC50 | T4 | - | - | 65 | 1.3 | 43.5 | 43065 | 33127 |
| PC51 | T4 | - | - | 66 | 2.2 | 47.5 | 12623 | 5738 |
| PC52 | T4 | - | - | 67 | 1.25 | 46.9 | 9653 | 7722 |

Supplementary Table 2. Serum samples used for DNA extraction. * Extraction efficiency was estimated based on ddPCR targeting the CPP1 spike-in.

| **PC samples** | **RP samples in**  **sample set 1 (n=20)** | **RP samples in**  **sample set 2 (n=19)** | **RP samples in**  **TCGA (n=498)** | **RP samples in**  **TCGA for survival analysis (n=392)** |
| --- | --- | --- | --- | --- |
| Age, years |  |  |  |  |
| Median (range) | 60 (50 -72) | 63 (54-73) | 61 (41-78) | 61 (43-78) |
| Pathological Gleason Score |  |  |  |  |
| <7, n (%) | 2 (10.0) | 7 (36.8) | 86 (17.3) | 70 (17.9) |
| 7, n (%) | 13 (65.0) | 8 (42.1) | 240 (48.2) | 187 (47.7) |
| >7, n (%) | 5 (25.0) | 4 (21.1) | 170 (34.1) | 133 (33.9) |
| Unknown, n (%) |  |  | 2 (0.4) | 2 (0.5) |
| Pathological T-stage |  |  |  |  |
| T2, n (%) | 11 (55.0) | 6 (31.6) | 188 (37.8) | 145 (37.0) |
| T3, n (%) | 8 (40.0) | 13 (68.4) | 293 (58.8) | 235 (59.9) |
| T4, n (%) | 1 (5.0) | 0 (0.0) | 10 (2.0) | 7 (1.8) |
| Unknown, n (%) | 0 (0.0) | 0 (0.0) | 7 (1.4) | 5 (1.3) |
| Pre-operative PSA |  |  |  |  |
| < 10 ng/mL, n (%) | 7 (35.0) | 4 (21.1) | 330 (66.3) | 262 (66.8) |
| ≥ 10 ng/mL, n (%) | 13 (65.0) | 15 (78.9) | 153 (30.7) | 117 (29.8) |
| Unknown, n (%) | 0 (0.0) | 0 (0.0) | 15 (3.0) | 13 (3.3) |
| Surgical margin status |  |  |  |  |
| Negative , n (%) | 11 (55.0) | 15 (78.9) | 316 (63.5) | 253 (64.5) |
| Positive, n (%) | 9 (45.0) | 4 (21.1) | 152 (30.5) | 123 (31.4) |
| Unknown, n (%) | 0 (0.0) | 0 (0.0) | 30 (6.0) | 16 (4.1) |
| Follow-up |  |  |  |  |
| PSA recurrence, n (%) | 0 (0.0) | 0 (0.0) | - | 46 (11.7) |
| No PSA recurrence, n (%) | 0 (0.0) | 0 (0.0) | - | 346 (88.3) |
| Unknown | 20 (100%) | 19 (100%) | - | 0 (0.0) |
| Follow-Up, months  Median (range) | - | - | - | 20.4 (3.0-153.5) |
| **Benign samples** | **NM samples in sample set 1 (n=21)** | **AN samples in sample set 2 (n=21)** | **AN samples in TCGA (n=67)** | **-** |
| Median Age in years (range) | 63 (48-80) | 61 (57-71) | 61 (43-72) | - |

Supplementary table 3: Clinicopathological data for patient sample sets analyzed on the 450K array for DNA methylation.

|  | Illumina ID | Chr | Position hg19 | Genomic element | Median benign (range) | Median PC (range) | Δβ | P-value benign vs. PC | P-value benign vs. PC (corrected) |
| --- | --- | --- | --- | --- | --- | --- | --- | --- | --- |
| Sample set 1 | cg00534240 | 1 | 76540148 | Island | 0.01 (0.00-0.02) | 0.02 (0.00-0.18) | 0.01 | **<0.001** | **0.006** |
| Sample set 1 | cg15090083 | 1 | 76540168 | Island | 0.02 (0.00-0.04) | 0.05 (0.02-0.55) | 0.03 | **<0.001** | **<0.001** |
| Sample set 1 | cg20707222 | 1 | 76540222 | Island | 0.06 (0.02-0.21) | 0.35 (0.08-0.57) | 0.29 | **<0.001** | **<0.001** |
| Sample set 1 | cg26363196 | 1 | 76540236 | Island | 0.08 (0.04-0.13) | 0.21 (0.08-0.38) | 0.13 | **<0.001** | **<0.001** |
| Sample set 1 | cg06211837 | 1 | 76540438 | Island, exon 1 | 0.02 (0.00-0.04) | 0.29 (0.06-0.66) | 0.27 | **<0.001** | **<0.001** |
| Sample set 1 | cg12601757 | 1 | 76540465 | Island, exon 1 | 0.07 (0.04-0.14) | 0.39 (0.14-0.64) | 0.32 | **<0.001** | **<0.001** |
| Sample set 1 | cg21526205* | 1 | 76540574 | Island, intron | 0.03 (0.01-0.23) | 0.56 (0.13-0.78) | 0.53 | **<0.001** | **<0.001** |
| Sample set 1 | cg27538686 | 1 | 76540641 | Island, intron | 0.06 (0.01-0.18) | 0.41 (0.21-0.58) | 0.35 | **<0.001** | **<0.001** |
| Sample set 2 | cg00534240 | 1 | 76540148 | Island | 0.01 (0.01-0.02) | 0.03 (0.01-0.16) | 0.02 | **<0.001** | **0.004** |
| Sample set 2 | cg15090083 | 1 | 76540168 | Island | 0.04 (0.03-0.07) | 0.11 (0.04-0.53) | 0.07 | **<0.001** | **<0.001** |
| Sample set 2 | cg20707222 | 1 | 76540222 | Island | 0.13 (0.06-0.29) | 0.34 (0.03-0.57) | 0.21 | **0.008** | **0.041** |
| Sample set 2 | cg26363196 | 1 | 76540236 | Island | 0.13 (0.10-0.22) | 0.27 (0.10-0.51) | 0.14 | **0.004** | **0.030** |
| Sample set 2 | cg06211837 | 1 | 76540438 | Island, exon 1 | 0.05 (0.03-0.27) | 0.35 (0.02-0.59) | 0.30 | **<0.001** | **0.003** |
| Sample set 2 | cg12601757 | 1 | 76540465 | Island, exon 1 | 0.10 (0.08-0.27) | 0.39 (0.07-0.69) | 0.29 | **<0.001** | **0.004** |
| Sample set 2 | cg21526205* | 1 | 76540574 | Island, intron | 0.09 (0.05-0.40) | 0.59 (0.27-0.76) | 0.49 | **<0.001** | **<0.001** |
| Sample set 2 | cg27538686 | 1 | 76540641 | Island, intron | 0.11 (0.06-0.33) | 0.43 (0.09-0.63) | 0.32 | **<0.001** | **0.002** |
| TCGA | cg00534240 | 1 | 76540148 | Island | 0.01 (0.01-0.22) | 0.02 (0.01-0.46) | 0.01 | **<0.001** | **<0.001** |
| TCGA | cg15090083 | 1 | 76540168 | Island | 0.02 (0.01-0.51) | 0.04 (0.02-0.84) | 0.02 | **<0.001** | **<0.001** |
| TCGA | cg20707222 | 1 | 76540222 | Island | 0.07 (0.02-0.58) | 0.32 (0.02-0.86) | 0.25 | **<0.001** | **<0.001** |
| TCGA | cg26363196 | 1 | 76540236 | Island | 0.10 (0.05-0.46) | 0.25 (0.04-0.75) | 0.15 | **<0.001** | **<0.001** |
| TCGA | cg06211837 | 1 | 76540438 | Island, exon 1 | 0.02 (0.01-0.70) | 0.37 (0.01-0.89) | 0.35 | **<0.001** | **<0.001** |
| TCGA | cg12601757 | 1 | 76540465 | Island, exon 1 | 0.06 (0.03-0.76) | 0.39 (0.02-0.88) | 0.33 | **<0.001** | **<0.001** |
| TCGA | cg21526205* | 1 | 76540574 | Island, intron | 0.03 (0.01-0.76) | 0.55 (0.01-0.95) | 0.52 | **<0.001** | **<0.001** |
| TCGA | cg27538686 | 1 | 76540641 | Island, intron | 0.07 (0.03-0.60) | 0.45 (0.02-0.85) | 0.38 | **<0.001** | **<0.001** |

Supplementary table 4. *ST6GALNAC3* Illumina 450K DNA methylation array data for Sample set 1: 20 PC and 21 benign (9 N and 12 AN), Sample set 2: 19 PC and 11 AN, and TCGA: 497 PC and 50 AN.

Genomic element: CpG island annotation from Illumina and gene structure annotation from UCSC gene track in the UCSC genome browser. Δβ: Median β (PC) - median β (benign). P-values from Mann-Whitney U tests, corrected: P-values Bonferroni corrected for multiple testing. *CpG site used for analyses of diagnostic and prognostic potential. Significant p-values in bold.

|  | Illumina ID | Chr | Position hg19 | Genomic element | Median benign (range) | Median PC (range) | Δβ | P-value benign vs. PC | P-value benign vs. PC (corrected) |
| --- | --- | --- | --- | --- | --- | --- | --- | --- | --- |
| Sample set 1 | cg21771463 | 3 | 44626400 | Island | 0.09 (0.07-0.14) | 0.13 (0.06-0.39) | 0.04 | **<0.001** | **<0.001** |
| Sample set 1 | cg03340649 | 3 | 44626453 | Island | 0.04 (0.02-0.20) | 0.37 (0.02-0.64) | 0.33 | **<0.001** | **<0.001** |
| Sample set 1 | cg22598028* | 3 | 44626492 | Island, exon 1 | 0.07 (0.03-0.22) | 0.44 (0.03-0.84) | 0.37 | **<0.001** | **<0.001** |
| Sample set 1 | cg01139508 | 3 | 44626538 | Island, intron | 0.04 (0.00-0.10) | 0.19 (0.03-0.63) | 0.15 | **<0.001** | **0.002** |
| Sample set 1 | cg17536166 | 3 | 44626697 | Island, intron | 0.07 (0.03-0.21) | 0.36 (0.06-0.68) | 0.29 | **<0.001** | **<0.001** |
| Sample set 2 | cg21771463 | 3 | 44626400 | Island | 0.14 (0.10-0.20) | 0.20 (0.09-0.50) | 0.06 | 0.056 | 0.278 |
| Sample set 2 | cg03340649 | 3 | 44626453 | Island | 0.12 (0.05-0.31) | 0.31 (0.05-0.58) | 0.19 | **0.001** | **0.005** |
| Sample set 2 | cg22598028* | 3 | 44626492 | Island, exon 1 | 0.11 (0.05-0.29) | 0.41 (0.07-0.70) | 0.30 | **<0.001** | **0.002** |
| Sample set 2 | cg01139508 | 3 | 44626538 | Island, intron | 0.02 (0.01-0.18) | 0.18 (0.01-0.46) | 0.16 | **<0.001** | **0.003** |
| Sample set 2 | cg17536166 | 3 | 44626697 | Island, intron | 0.11 (0.08-0.22) | 0.35 (0.11-0.55) | 0.24 | **<0.001** | **0.001** |
| TCGA | cg21771463 | 3 | 44626400 | Island | 0.09 (0.06-0.47) | 0.26 (0.04-0.92) | 0.17 | **<0.001** | **<0.001** |
| TCGA | cg03340649 | 3 | 44626453 | Island | 0.03 (0.01-0.51) | 0.43 (0.01-0.88) | 0.40 | **<0.001** | **<0.001** |
| TCGA | cg22598028* | 3 | 44626492 | Island, exon 1 | 0.05 (0.02-0.61) | 0.45 (0.02-0.93) | 0.40 | **<0.001** | **<0.001** |
| TCGA | cg01139508 | 3 | 44626538 | Island, intron | 0.03 (0.02-0.57) | 0.13 (0.02-0.96) | 0.10 | **<0.001** | **<0.001** |
| TCGA | cg17536166 | 3 | 44626697 | Island, intron | 0.08 (0.04-0.48) | 0.36 (0.03-0.86) | 0.28 | **<0.001** | **<0.001** |

Supplementary table 5. *ZNF660* Illumina 450K DNA methylation array data for Sample set 1: 20 PC and 21 benign (9 N and 12 AN), Sample set 2: 19 PC and 11 AN, and TCGA: 497 PC and 50 AN. Genomic element: CpG island annotation from Illumina and gene structure annotation from UCSC gene track in the UCSC genome browser. Δβ: Median β (PC) - median β (benign). P-values from Mann-Whitney U tests, corrected: P-values Bonferroni corrected for multiple testing. *CpG site used for analyses of diagnostic and prognostic potential. Significant p-values in bold.

| Model | Genes | Parameters | BPH vs  PC |
| --- | --- | --- | --- |
| 1 Gene | *ST6GALNAC3* | AUC  Specificity  Sensitivity | 0.648  100%  30% |
| 1 Gene | *ZNF660* | AUC  Specificity  Sensitivity | 0.611  100%  22% |
| 1 Gene | *CCDC181* | AUC  Specificity  Sensitivity | 0.630  100%  26% |
| 1 Gene | *HAPLN3* | AUC  Specificity  Sensitivity | 0.722  100%  44% |
| 2 Genes | *ST6GALNAC3 / ZNF660* | AUC  Specificity  Sensitivity | 0.685  100%  37% |
| 2 Genes | *ST6GALNAC3 / CCDC181* | AUC  Specificity  Sensitivity | 0.759  100%  52% |
| 2 Genes | *ST6GALNAC3 / HAPLN3* | AUC  Specificity  Sensitivity | 0.796  100%  59% |
| 2 Genes | *ZNF660 / CCDC181* | AUC  Specificity  Sensitivity | 0.704  100%  41% |
| 2 Genes | *ZNF660 / HAPLN3* | AUC  Specificity  Sensitivity | 0.759  100%  52% |
| 2 Genes | *CCDC181 / HAPLN3* | AUC  Specificity  Sensitivity | 0.778  100%  56% |
| 3 Genes | *ST6GALNAC3 / ZNF660 / CCDC181* | AUC  Specificity  Sensitivity | 0.778  100%  56% |
| 3 Genes | *ST6GALNAC3 / ZNF660 / HAPLN3* | AUC  Specificity  Sensitivity | 0.796  100%  59% |
| 3 Genes | *ST6GALNAC3 / CCDC181 / HAPLN3* | AUC  Specificity  Sensitivity | **0.833**  **100%**  **67%** |
| 3 Genes | *ZNF660 / CCDC181 / HAPLN3* | AUC  Specificity  Sensitivity | 0.796  100%  59% |
| 4 Genes | *ST6GALNAC3 / ZNF660 / CCDC181 / HAPLN3* | AUC  Specificity  Sensitivity | **0.833**  **100%**  **67%** |

Supplementary table 6. AUCs, sensitivities and specificities of methylation versus no methylation in serum samples. Highest AUCs in bold with grey background.


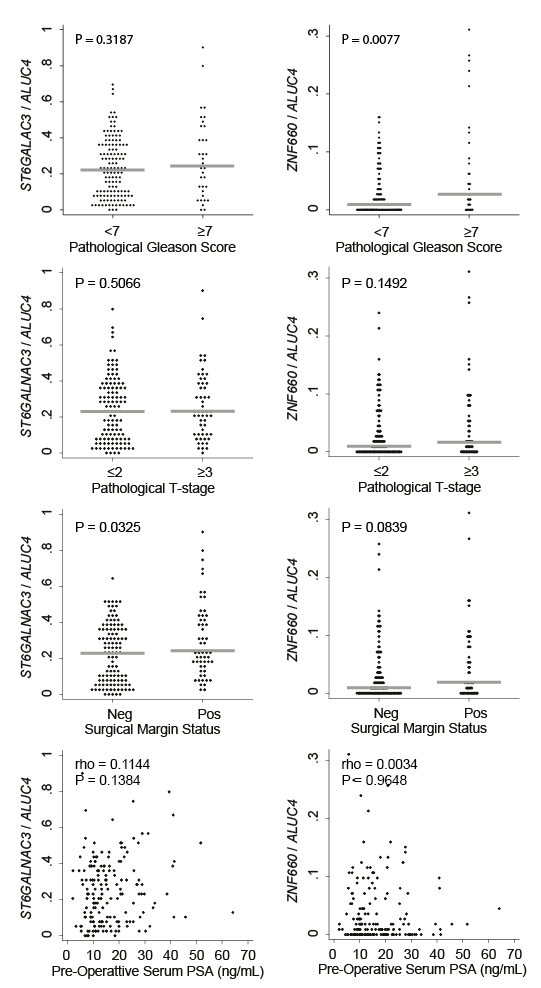


Supplementary Figure 1 - Correlation of promoter methylation, as determined by qMSP, to clinicopathological parameters in RP cohort 1 (n=169). Grey lines: median methylation. P-values from Mann-Whitney U tests for all comparisons, except PSA, where the Spearman correlation rho- and p-values are given.


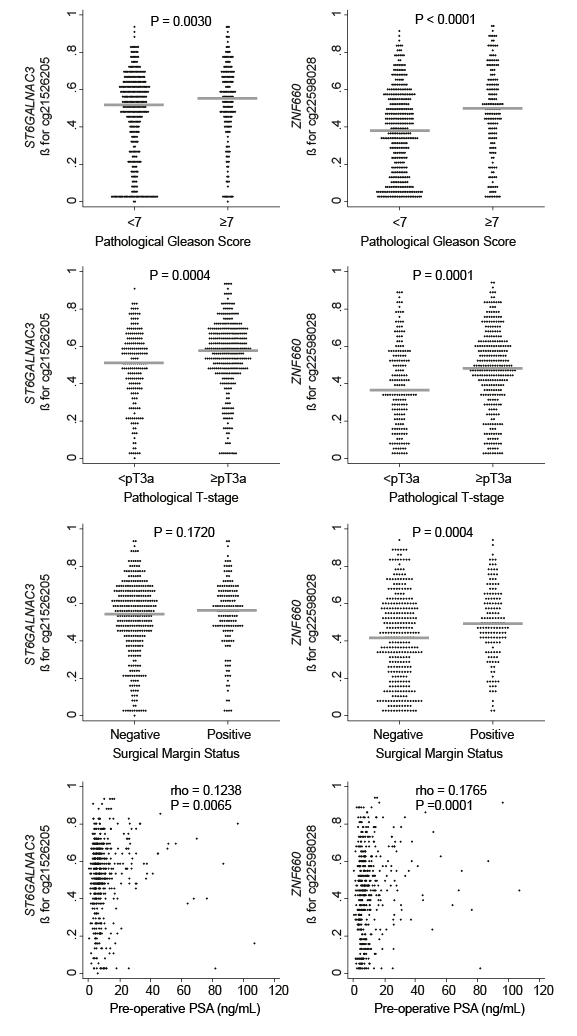


Supplementary Figure 2 - Correlation of promoter methylation, as determined by 450K array, to clinicopathological parameters in the TCGA RP cohort (n=497). Grey lines: median methylation. P-values from Mann-Whitney U tests for all comparisons, except PSA, where the Spearman correlation rho- and p-values are given.

**References**

Haldrup, C., Mundbjerg, K., Vestergaard, E.M., Lamy, P., Wild, P., Schulz, W.A., Arsov, C., Visakorpi, T., Borre, M., Hoyer, S., Orntoft, T.F., Sorensen, K.D., 2013. DNA methylation signatures for prediction of biochemical recurrence after radical prostatectomy of clinically localized prostate cancer. J Clin Oncol 31, 3250-3258.

Reinert, T., Scholer, L.V., Thomsen, R., Tobiasen, H., Vang, S., Nordentoft, I., Lamy, P., Kannerup, A.S., Mortensen, F.V., Stribolt, K., Hamilton-Dutoit, S., Nielsen, H.J., Laurberg, S., Pallisgaard, N., Pedersen, J.S., Orntoft, T.F., Andersen, C.L., 2016. Analysis of circulating tumour DNA to monitor disease burden following colorectal cancer surgery. Gut 65, 625-634.

Weisenberger, D.J., Campan, M., Long, T.I., Kim, M., Woods, C., Fiala, E., Ehrlich, M., Laird, P.W., 2005. Analysis of repetitive element DNA methylation by MethyLight. Nucleic Acids Res 33, 6823-6836.
